# The Mediating Role of Meaning-Making in the Relationship Between Mental Time Travel and Positive Emotions in Stress-Related Blogs: Big Data Text Analysis Research

**DOI:** 10.2196/63407

**Published:** 2025-02-21

**Authors:** Yidi Chen, Lei Zheng, Jinjin Ma, Huanya Zhu, Yiqun Gan

**Affiliations:** 1 School of Humanities and Social Sciences Beijing Forestry University Beijing China; 2 School of Business and the Institute for Sustainable Development Macau University of Science and Technology Macau China; 3 School of Psychological Cognitive Sciences, and Beijing Key Laboratory of Behavior and Mental Health Peking University Beijing China; 4 School of Psychological Cognitive Sciences, and Key Laboratory of Machine Perception (Ministry of Education) Peking University Beijing China

**Keywords:** stress, meaning-making, mental time travel, big data, mini meta-analysis, text analysis, coping mechanisim, Weibo, post, web crawler, positive emotion, emotion, meta-analysis, anxiety, depression, mental health, ecological momentary assessment, EMA, stress model, natural language processing, NLP

## Abstract

**Background:**

Given the ubiquity of stress, a key focus of stress research is exploring how to better coexist with stress.

**Objective:**

This study conducted text analysis on stress-related Weibo posts using a web crawler to investigate whether these posts contained positive emotions, as well as elements of mental time travel and meaning-making. A mediation model of mental time travel, meaning-making, and positive emotions was constructed to examine whether meaning-making triggered by mental time travel can foster positive emotions under stress.

**Methods:**

Using Python 3.8, the original public data from active Weibo users were crawled, yielding 331,711 stress-related posts. To avoid false positives, these posts were randomly divided into two large samples for cross-validation (sample 1: n=165,374; sample 2: n=166,337). Google’s natural language processing application programming interface was used for word segmentation, followed by text and mediation analysis using the Chinese psychological analysis system “Wenxin.” A mini–meta-analysis of the mediation path coefficients was conducted. Text analysis identified mental time travel words, meaning-making words, and positive emotion words in stress-related posts.

**Results:**

The constructed mediation model of mental time travel words (time words), meaning-making words (causal and insightful words), and positive poststress emotions validated positive adaptation following stress. A mini–meta-analysis of two different mediation models constructed in the two subsamples indicated a stable mediation effect across the 2 random subsamples. The combined effect size (B) obtained was .013 (SE 0.003, 95% CI 0.007-0.018; *P*<.001), demonstrating that meaning-making triggered by mental time travel in stress-related blog posts can predict positive emotions under stress.

**Conclusions:**

Individuals can adapt positively to stress by engaging in meaning-making processes that are triggered by mental time travel and reflected in their social media posts. The study’s mediation model confirmed that mental time travel leads to meaning-making, which fosters positive emotional responses to stress. Mental time travel serves as a psychological strategy to facilitate positive adaptation to stressful situations.

## Introduction

### Stress and Mental Health

Researchers have consistently linked stressful events to negative emotions and adverse psychological health outcomes, viewing stress as an inevitable precursor of anxiety, depression, and other negative emotional states [1,2]. A recent meta-analysis of ecological momentary assessments suggested a significant positive correlation between transient negative emotions and cortisol levels—a biomarker of stress—whereas positive emotions were significantly and negatively correlated with cortisol levels [3].

The cumulative stress model posits that the negative impact of stress on an individual’s emotions and psychological health is cumulative, emphasizing the severe and lasting effects of early life stress on the brain [4-6]. Prolonged exposure to stress can also diminish a person’s ability to withstand future stressors, thereby impacting mental health [7]. Stress has been shown to negatively impact memory, executive function, emotional regulation, and the processing of social and emotional stimuli [8]. Chronic exposure to stress could lead to neurodevelopmental changes in the brain structure and function [9].

These findings and the cumulative stress model suggest that experiencing stressful events is detrimental to individuals, and avoiding such events is beneficial for mental health. However, these studies only emphasize individual vulnerability, overlooking psychological resilience and the potential for growth and development in the face of adversity. The control and mastery theory of anxiety suggests that effective coping with stressful events can enhance an individual’s sense of control and better prepare them for future stressors [10]. The concept of “stress inoculation” also supports the idea that “what does not kill us makes us stronger” [11]. Recent research evidence indicates that over 60% of individuals demonstrate adaptive functioning after experiencing stressful events or adversity, commonly referred to as “stress resilience” [12].

The cyclical model of stress resilience and stress-related growth posits that the relationship between stress and mental health outcomes such as positive emotions is not necessarily negative [13-17]. After a stressful event, individuals initiate an adjustment process that leads to psychological outcomes of the stress event. The nature of the adjustment process and its outcomes depend on the severity of the stress experienced. If the severity of a stressful event is within an individual’s tolerance, successful adjustment could enhance resilience and lead to positive coping outcomes. Events causing minimal stress could require little adjustment and are likely to lead to the recovery and restoration of resilience to baseline levels [18-20]. Highly stressful events can overwhelm an individual’s resilience and cause damage, at least temporarily. The level of resilience is modulated by various factors, of which meaning-making is a crucial coping mechanism [13,18,21-24].

### Mental Time Travel to Trigger Meaning-Making

Meaning-making is a stress-coping strategy aimed at altering an individual’s appraisal of a situation to harmonize their beliefs, goals, and stress context. Unlike classic problem- and emotion-focused coping strategies, meaning-making does not attempt to change problematic situations or directly reduce negative emotions or pain [25]. Instead, it unifies beliefs, goals, and stress contexts by searching for meaning [26].

The meaning-making model proposed and revised by Park [27] is a widely accepted theory for understanding how individuals cope positively with stressful events and situations. It centers on the concept of “global meaning”—a cognitive framework that individuals use to interpret their experiences and motivations [25]. The model posits that when individuals encounter situations that might challenge or reinforce this global meaning, they engage in an evaluative process that assigns significance to these situations. The degree of discrepancy between the appraised meaning and its inherent global meaning determines the level of distress experienced. This distress initiates the process of meaning-making, wherein individuals strive to reconcile the gap between situational meaning and global understanding. This reconciliation effort aims to restore a sense of coherence in the world and reaffirm the value of their lives. Ultimately, achieving harmonization between situational and global meanings facilitates better adaptation to stress, highlighting the transformative role of meaning-making in managing life challenges [28-32].

In laboratory studies, two common methods are often used to elicit meaning-making in individuals: mental time travel and meaning-threat paradigms. Among these, mental time travel, a type of typical mental travel that predominantly involves recalling the past or imagining the future, is the most frequently used. Mental time travel is the ability to project oneself into the past or future through mental time travel [33]. As previously stated, individuals were said to experience mental time travel when they re-experienced events from their past and imagined events in their future [34,35]. Construct level theory suggests that when individuals remember the past or think about the future, they process things at a more abstract and superordinate level because temporal distance causes the mental distance to become farther away [36]. This is consistent with the findings of research in the area of mental time travel and the process of meaning-making and meaning-acquisition [37]. Research suggests that coherence is an important component of meaning in life, which involves an understanding of personal experiences and a sense of life events [38]. Through mental time travel, people recall past events or imagine future events, which helps them integrate their life experiences from different times into a more cohesive meaning and enhances their self of purpose in life [39]. Numerous studies indicate that mental time travels in the form of nostalgia or future contemplation can enhance the meaning in life or positive emotions owing to the role of meaning-making [40-42]. During stressful situations, individuals initiate the process of meaning-making; seek clues from the past, present, or future; integrate life experiences to redefine the current stress context; or adjust their global meaning to guide their future lives [43]. Thus, when individuals attempt to mentally travel to the past or future, they can enhance positive emotions and predict higher psychological well-being through meaning-making [27].

Experimental studies and research into cognitive neural mechanisms have also found evidence that mental time travel successfully initiates meaning-making. In one laboratory study, participants were randomly assigned to future, present, and past groups and asked to imagine 2 independent events within different timeframes. Participants imagining the future and past reported higher meaning in life than those imagining the present. This indicates that mental time travel, through self-distancing from the here and now, promotes the integration of temporal information and causal understanding, becoming a stable paradigm for eliciting meaning-making [44-46]. In a functional magnetic resonance imaging study using a similar mental time-travel paradigm to successfully elicit meaning-making, a decrease in default mode network activity was observed, linking meaning-making with positive psychological health outcomes [47].

### This Study

**Figure 1:**
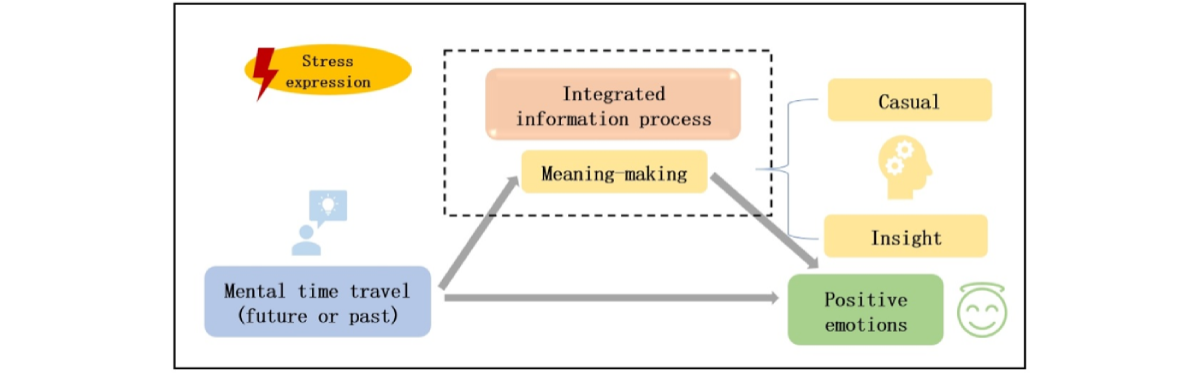
The hypothesis model.

The internet is now an integral part of people’s lives and the rapid development of social media has significantly altered how people express their emotions [48]. The evolution of the internet and big data has propelled psychological research. In Chinese social media, Sina Weibo has become a crucial data source for psychological studies owing to its fast dissemination, timely information, and wide audience [49]. When individuals perceive stress, they may express their emotions or reflect by posting on Sina Weibo. The cyclical model of stress resilience and stress-related growth posits that stress has an adaptive function that is facilitated by the coping process [50]. Research indicates that expressive inhibition is a maladaptive coping strategy and a possible mechanism for affect dysregulation following trauma [51]. In contrast, expressive writing during stressful situations has been shown to relieve stress and hone coping strategies, thereby alleviating negative emotions and promoting positive adaptation [52]. This process can also be accomplished through mental time travel, which facilitates the process of meaning-making by enabling individuals to integrate insights about the meaning of life from the past and future, thus promoting positive adaptation following stress [43]. Therefore, a theoretical model that comprises mental time travel and the cyclical model of stress resilience and stress-related growth is proposed in this study. This study explored whether such actions entail a process of meaning-making, such as causal connections or insights, and whether successful meaning-making is followed by positive adaptation to stress, manifested as positive emotions (Figure 1).

Specifically, this research involved text analysis of stress-related blog posts on Weibo to investigate the presence of positive elements as indicators of poststress adaptation and to explore elements of meaning-making in everyday stress and coping. Previous studies have frequently used mental time travel as a stable paradigm to elicit meaning-making, often inviting participants to imagine past or future events [44-46]. Mental time travel can reliably initiate meaning-making for individuals, thereby enhancing meaning in their lives. The words “future” and “past” are used as indicators of mental time travel, and causal and insightful words serve as key components of the meaning-making process. This study examined whether the meaning-making process mediates the impact of mental time travel on positive emotions. In this study, words related to past and future blog posts were considered components of mental time travel that triggered meaning-making. Emotional words were used to represent positive outcomes under stress, whereas words referring to the past or future served as markers of mental travel. In qualitative research and text analysis, insightful words (eg, “originally” and “comprehend”) and causal words (eg, “because,” “therefore,” and “thus”) were often used as components of meaning-making [53]. Thus, these words were used as indicators of meaning-making. Building on this, a mediation model of mental time travel, meaning-making, and positive emotions poststress was constructed, and a mini–meta-analysis was used to test whether mental time travel-induced meaning-making can promote positive emotions under stress.

Meta-analysis is a data analysis method that reanalyzes multiple independent quantitative studies on the same question to derive more generalizable conclusions [54]. Recently, statisticians have recognized that meta-analyses do not necessarily require several studies, and even two effect sizes can suffice. In fact, even with a few effect sizes, meta-analysis can summarize them, not only clarifying whether the research question has been consistently answered but also enhancing the persuasiveness of the conclusions, especially when the detected effect sizes are small. Individual studies may lack sufficient evidence, but collectively, they can be compelling. Researchers suggest that even a limited number of studies (or their subgroups) can be compared with each other. Such an internal meta-analysis of conceptually comparable studies is known as a mini–meta-analysis [55].

This study aimed to explore the mediating role of meaning-making in the relationship between mental time travel and positive emotions in stress-related blogs. In this study, a mini–meta-analysis was performed to detect the mediation model.

## Methods

### Research Objects

This study was conducted using original posts from active microblog users, excluding accounts belonging to institutions and media outlets. The screening criteria were as follows: (1) a publicly published blog by an individual user, (2) the blog was posted between December 2019 and November 2020, and (3) the blog post included the keyword ‘‘stress.” All blog posts have been anonymized. A statistical analysis was performed, revealing 331,711 posts related to “stress.” Given the propensity of big data to yield false positives, these posts were split into two random samples using the random grouping function in SPSS (version 24.0; IBM Corp). This division facilitated cross-validation of data reliability. Ensuing analyses were conducted on these two substantial samples (sample 1: n=165,374; sample 2: n=166,337).

### Ethical Considerations

This study was approved by the School of Psychological and Cognitive Science, Peking University Ethics Committee (#2018-10-03).

### Design and Programming

The Sina Weibo blog posts were harvested using Python 3.8, using the “lxml,” “requests,” and “tqdm” libraries to acquire and process HTML page data. This method involves retrieving key data such as the source, ID, content of the blog, date, and metrics, including the number of likes, comments, and reposts. All retrieved data were then systematically stored for further analysis.

### Data Analysis

Google’s natural language processing application programming interface was used for word segmentation. Following this, a dictionary based on the Linguistic Inquiry and Word Count (LIWC) framework was used to conduct a word frequency analysis. This process enabled the identification of high-frequency words and their respective counts in the blog posts. Subsequently, the Chinese psychological analysis system “Wenxin”—developed by the Computational Network Psychology Laboratory at the Institute of Psychology, Chinese Academy of Sciences—was applied to analyze the content of the blog posts. “Wenxin” is a sophisticated software system designed for the linguistic analysis of Chinese texts. It features automatic word separation and text analysis capabilities, leveraging both LIWC2007 and the orthographic Chinese C-LIWC lexicon, which is tailored to the linguistic context of simplified Chinese in mainland China [56]. The “Wenxin” system uses lexicon technology to conduct word segmentation, extract word classes, perform word frequency analysis, and calculate the proportion of mental time travel, meaning-making, and positive emotion words within the entire blog post. During the process, “happy” is generally considered a word signifying positive emotion, while “not happy” would be classified as a word signifying negative emotion. In order to prevent large sample false positives, we used random grouping to randomly divide the sample into two subsamples for cross-validation (Koul et al [57]). Correlation and mediation analyses were conducted using SPSS version 24.0. During mediation analyses, path coefficients and effect sizes (B and SE) were extracted and stored in a dataset. First, using the SPSS 24.0 macro file PROCESS [58], a bootstrapping procedure was used to verify whether mental time travel words (future and past time words) could mediate the effect of meaning-making words (causal words, eg, because, effect, hence, and insightful words, eg, think, know, consider; see Table S5 in Multimedia Appendix 1) on positive emotions under stress [56,59]. Model 4 was selected by setting the number of samples to 5000 and using a bias-corrected 99% CI. To control for the type I error inflation caused by multiple comparisons, the significance level (α) of correlations was adjusted to .01 and the CI of the mediating effect was adjusted to 99% with Bonferroni correction [60].

Mediation heterogeneity tests, composite effect size calculations, and publication bias tests were performed using the “metafor” package in R (R Foundation and R Caucus). For visualization purposes, the “forestplot” function was used. A meta-analysis was conducted to verify the stability and homogeneity of these effects across the samples. The first step was to perform a Q-test for homogeneity to assess the similarity between multiple independent studies. If the homogeneity test was not significant and *I*^2^ was <40%, the criteria for homogeneity were met, and a fixed-effects model was used to estimate the combined effect size. Conversely, if the homogeneity test was significant, a random-effects model was used.

## Results

In the retrieved blog posts, people’s stressful events were concentrated in the areas of work stress (eg, working all day at the office with thunder outside), life stress (eg, pressure to buy and rent), academic stress (eg, pressure to graduate), family stress (eg, babysitting), and intimate relationships (eg, finding a partner). By leveraging the outcomes of Google Segmentation and Word Frequency Analysis, we derived insights into sentiment tones and word frequency in blog posts concerning stress and meaning-making strategies. The analysis of these charts revealed that in stress-related blog posts, prevalent themes included personal references such as “self,” “we,” and “they.” Additionally, there was a significant presence of cause-and-effect related terms like “reason,” “so,” and “because.” These findings underscore the process of meaning-making. There were also time-related elements such as “one day” and “tomorrow” (Table 1).

**Table 1:** Word frequency table of high-frequency words for word frequency analysis of microblog posts (top 60).

No.	Text	Frequency
1	Self	4491
2	No	2024
3	We	1692
4	Reason	1587
5	Livelihood	1535
6	Can	1439
7	Moment	1326
8	Work	1230
9	Feel	1190
10	Now	1179
11	Because	1136
12	Don’t	1084
13	But	1066
14	This	970
15	Today	951
16	Hope	943
17	Feelings	909
18	Recently	869
19	How	829
20	Happy	764
21	If	762
22	Time	760
23	May	759
24	Try	745
25	Everyone	737
26	So	725
27	They	712
28	Thing	708
29	Other	692
30	Everyday	682
31	Actually	669
32	Won’t	666
33	Many	666
34	Together	657
35	Good night	652
36	Always	649
37	Such	636
38	Already	635
39	Start	626
40	Like	614
41	Kid	607
42	A little	598
43	Big	578
44	Happiness	569
45	Question	548
46	Activity	547
47	And	535
48	Can’t	531
49	Friend	507
50	Don’t know	496
51	Tomorrow	491
52	Fighting	490
53	See	470
54	Need	468
55	One day	468
56	Although	464
57	Know	463
58	Withstand	460
59	Emotion	447
60	Life	438

In this meaning-making process, there are initial attributions and causal understandings, followed by the emergence of new attributions in ongoing meaning-making. We found that recalling the past and contemplating the future are accompanied by a process of meaning-making. For example, “Thanks to my past self for staying true to myself when the pressure was on. Thanks for the self that still wipes away the tears and memorizes after every cry. Every step I took, every tear I left behind, the pain I felt when I was suffering. All are the marks of youth. In the future, I will thank myself for struggling now.” “Recently, I have been under a lot of pressure, and I have drawn up a distant travel plan for myself. Winter vacation to Finland to see reindeer, and want to drink hot horse milk. Thinking about it makes me feel healed to the point where I wish I could kill all the malice and love is worth it.” This example illustrates the meaning-making process triggered by mental time travel. Under pressure, people can make meaning by expressing themselves on blogs. For example, “There are many things in this world that can make you happy, one long-term and one short-term. It’s up to you, what you are, how much stress you can handle, and how much better and longer-term benefits you can enjoy.” “Recently, you needed to organize yourself, organize your emotions, organize your life, organize your work, organize your plans for the future, always have to do a good job of planning, in order to have peace of mind, there is pressure to continue to move forward, I believe that everything can be better and better.” “When you navigate the difficulties and obstacles alone, you will be grateful for the first time to say nothing and bite your teeth to insist on your own path.” “Still young, not everything in life is decided over a small thing, pressure will exist, but it won’t last forever or learn to be more optimistic and happier.” “No pressure of life will be empty, no pressure of youth will wither, no pressure of life will be bleak.”

Subsequently, to delve deeper into how the components of meaning-making in stress-related blog posts contribute to the positive adaptation to stress, the “Wenxin” system was used for textual analysis. This analysis involved extracting elements related to time, meaning-making, and positive or negative emotions. The frequencies and percentages of these elements were included in the subsequent mediation analyses. For the time elements, components representing the past, present, and future were selected. For meaning-making, words signifying attribution and causal understanding were chosen, such as causal words (eg, “because,” “therefore,” and “thus”) and insightful words (eg, “originally” and “comprehend”). Positive emotional words were counted based on their frequency. The descriptive statistics and correlation analyses for the two large samples are presented in Tables 2 and 3.

**Table 2:** Descriptive statistics and correlation analysis of main variables for sample 1 text analysis (n=165,374).

		Percentage values, mean (SD)	1	2	3	4	5	6
1	Rate of positive emotions words	0.10 (0.004)	1.00					
2	Rate of insightful words	0.06 (0.002)	0.07^a^	1.00				
3	Rate of causal words	0.03 (0.001)	0.03^a^	0.10^a^	1.00			
4	Rate of past time words	0.01 (0.002)	0.01^b^	0.03^a^	0.01^a^	1.00		
5	Rate of present time words	0.01 (0.001)	0.02^a^	0.03^a^	0.07	0.03^a^	1.00	
6	Rate of future-time words	0.01 (0.001)	0.03^a^	0.01^a^	–0.01	0.01^b^	0.03^a^	1.00

^a^*P*<.001.

^b^*P*<.01.

**Table 3:** Descriptive statistics and correlation analysis of main variables for sample 2 text analysis (n=166,337).

		Percentage value, mean (SD)	1	2	3	4	5	6
1	Rate of positive emotions words	0.10 (0.004)	1.00					
2	Rate of insightful words	0.06 (0.019)	.07^a^	1.00				
3	Rate of causal words	0.03 (0.001)	.02^a^	.10^a^	1.00			
4	Rate of past time words	0.01 (0.003)	.01	.02^a^	.02^a^	1.00		
5	Rate of present time words	0.01 (0.007)	.01^b^	.02^a^	.01^a^	.04^a^	1.00	
6	Rate of future-time words	0.01 (0.005)	.02^a^	.03^a^	–.01	.01	.04^a^	1.00

^a^*P*<.001.

^b^*P*<.01.

Correlation analyses of the 2 large samples revealed significant positive correlations between the frequency of positive emotion words and the frequency of causal (sample 1: *r*=0.03, *P*<.001; sample 2: *r*=0.02, *P*<.001) and insightful (both sample: *r*=0.07, *P*<.001) words (meaning-making). In sample 1, positive emotion words showed a significant positive correlation with the rates of future-time words (*r*=0.03; *P*<.001), past-time words (*r*=0.01; *P*=.005), and present-time words (*r*=0.02; *P*<.001). In sample 2, there was a significant positive correlation between positive emotion words and the frequencies of both present (*r*=0.01; *P*=.005) and future words (*r*=0.02; *P*<.001). The frequencies of causal (sample 1: *r*=0.01, *P*<.001; sample 2: *r*=0.02, *P*<.001) and insightful words (sample 1: *r*=0.03; *P*<.001; sample 2: *r*=0.02, *P*<.001) were positively correlated with past words, but future words were only significantly positively correlated with insightful words (sample 1: *r*=0.01, *P*<.001; sample 2: *r*=0.03, *P*<.001) and not causal words (*P*>.01 in both samples). This aligns with the notion that attributions are more prevalent when reflecting on the past than the future.

Mediation models were established for both samples (Table 4).

In this study, the results of the homogeneity test were *Q*_1_= 0.93 and *P*=.34, with an *I*^2^ of 0 %, indicating that the criteria for homogeneity were met. Therefore, a fixed-effects model was chosen for the analysis.

**Table 4:** Mediation models.

Sample	Mental time travel	Meaning-making	Positive emotions	a path^a^, B	b path^b^, B	c path^c^, B	Indirect effect	99% CI
1	Rate of future-time words	Rate of insightful words	Rate of positive emotions words	.04^d^	.17^d^	.23^d^	0.008	0.002 to 0.021
2	Rate of future-time words	Rate of insightful words	Rate of positive emotions words	.10^d^	.17^d^	.14^d^	0.017	0.007 to 0.037
1	Rate of future-time words	Rate of causal words	Rate of positive emotions words	–.01	.11^d^	.24^d^	–0.002	–0.005 to –0.001
2	Rate of future-time words	Rate of causal words	Rate of positive emotions words	–.01	.07^d^	.16^d^	–0.001	–0.002 to 0.001
1	Rate of past time words	Rate of insightful words	Rate of positive emotions words	.18^d^	.17^d^	.08^e^	0.030	0.016 to 0.052
2	Rate of past time words	Rate of insightful words	Rate of positive emotions words	.11^d^	.17^d^	–.01	0.019	0.007 to 0.041
1	Rate of past time words	Rate of causal words	Rate of positive emotions words	.05^d^	.11^d^	.10^f^	0.005	0.001 to 0.025
2	Rate of past time words	Rate of causal words	Rate of positive emotions words	.09^d^	.07^d^	.01	0.006	–0.001 to 0.025

^a^Indicates the path from mental time travel to meaning-making.

^b^Indicates the path from meaning-making to positive emotions.

^c^Indicates the path from mental time travel to positive emotions.

^d^*P*<.001.

^e^*P*<.05.

^f^*P*<.01.

**Figure 2:**
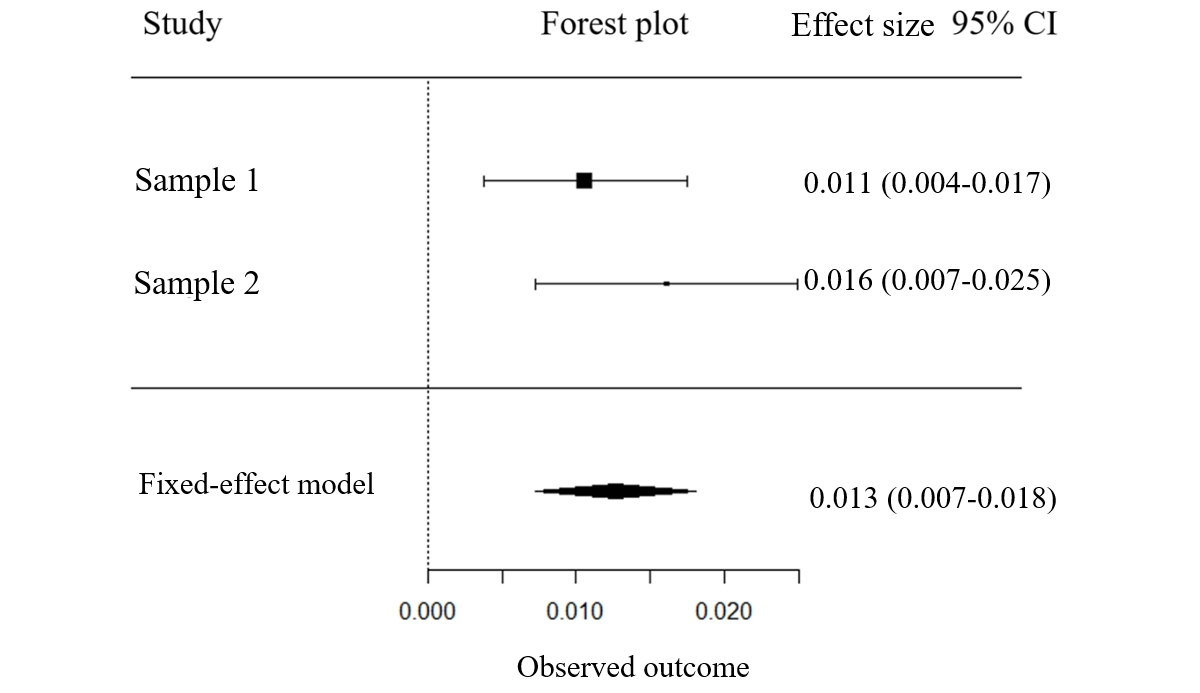
Forest plot illustrating the mediation effect of mental time travel-meaning-making on positive emotions.

After extracting each effect size to be included in the meta-analysis, the values were synthesized and weighted to calculate a combined average statistic. The calculation of the average effect size was performed by weighting each effect size according to the inverse of its variance (ϖi). This involved multiplying each effect size by its respective weight, summing the products, and dividing them by the sum of the weights. Next, the standard error of the mean was calculated using the square root of the sum of the inverse variance weights, 
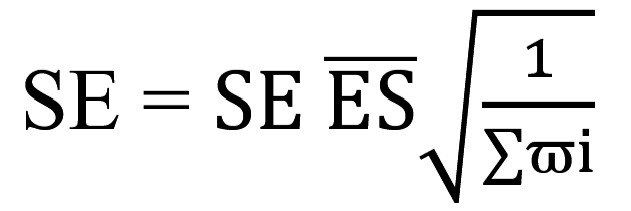
. This standard error was used to determine 95% CIs. The combined effect size (B) obtained was .013 (SE 0.003; 95% CI 0.007-0.018; *P*<.001; Figure 2).

## Discussion

### Principal Findings

This study conducted a large-scale text analysis of stress-related Weibo posts to identify the coexistence of positive emotional words and stress. Meaning-making elements were also observed in these posts. This study found that meaning-making induced by mental time travel can promote positive emotions under stress. The presence of words related to the past and future reflects the mental travel time. Recognizing that life encompasses not only the present but also the past and future is a prerequisite for meaning-making through complete narratives. Therefore, the appearance of future and past words signifies a meaning-making process. Causal and insightful words representing the outcomes of meaning-making also appeared in the posts. This discovery is significant, as a wealth of research evidence indicates that mental time travel initiates the meaning-making process and concurrently influences shifts in one’s sense of meaning in life [40-42]. Therefore, this analysis provides a nuanced understanding of how individuals engage and narrate their experiences of stress and coping within a digital context.

A mediation model was constructed using mental time travel words (mental time travel words) → meaning-making words (causal and insightful words) → positive poststress emotions. This model confirmed positive adaptation following stress. Mini–meta-analyses of two combined mediation models tested in 2 subsamples revealed a stable mediation effect across the 2 random subsamples. This demonstrates that meaning-making induced by mental time travel in stress-related blog posts can predict positive emotions under stress, supporting the cyclical model of stress resilience and stress-related growth rather than the cumulative stress model. This finding contributes to addressing the decade-long imbalance in stress research and clinical practice, which has almost exclusively focused on negative emotions while neglecting positive ones [61].

### The Adaptive Function of Meaning-Making

The stress interaction model indicates that stress does not necessarily lead to impairment or maladaptation. An individual’s diverse experiences in response to the same stressor primarily stem from their primary and secondary appraisals of the stressor, with meaning-making playing a critical role [62]. The meaning-making model further explains how meaning-making reduces the distress caused by a stressor and fosters growth by narrowing the discrepancy between situational and global meanings [27].

The cyclical model of stress resilience and stress-related growth posits that the initial state of individuals is determined by a combination of genetic, environmental, and social factors. When faced with stress, individuals adjust, leading to stress coping, which becomes a new initial state. During this adjustment process, certain coping strategies and personal resources can facilitate positive poststress psychological health outcomes, with meaning-making being an effective strategy [50]. Both meaning-making and cyclical models of stress resilience and growth indicate that coping strategies, such as meaning-making, can serve as mediating mechanisms leading to positive outcomes related to stress; hence, meaning-making can promote positive emotions under stress [27,50].

Research in the field of positive psychology has shown that meaning-making reliably predicts an individual’s psychological health [63,64]. Extensive literature has demonstrated the adaptive function of meaning-making, showing a consistent positive correlation between meaning-making and positive psychological outcomes [61,65,66]. A meta-analysis of 87 cross-sectional studies found that meaning-making was associated with greater happiness and less depression [66]. In older adults living in nursing facilities, meaning-making was significantly correlated with positive emotions, personal growth, environmental mastery, and self-acceptance [67]. In a study of adolescent earthquake survivors, meaning-making was related to posttraumatic growth, which explains the relationship between meaning-focused coping and positive emotions [68].

### Benefits of Mental Time Travel

Meaning-making involves the integration of an individual’s life experiences, a process that includes narrative elements [27]. This means that individuals attempt to find common threads across their past, present, and future experiences, creating coherent and understandable cognitive representations or schema. Meaning-making requires the recognition that life transcends any specific moment, and mental time travel possesses these narrative elements. Mental time travel, also known as self-distancing or mental simulation, is the process of mentally transcending oneself. It involves occupying different times (past or future) or places or assuming the subjective experiences of different people or hypothetical realities. Mental time travel encompasses imagining experiences that extend beyond what is currently in progress. This capacity for simulation, which is particularly rich in form, seems unique to humans [69]. Some scholars have speculated that simulation enables humans to engage in complex cultures by navigating past, future, and social worlds [70]. A tendency toward nostalgia can mitigate the impact of existential threats on one’s sense of meaning and increase positive emotions [40,41]. Additionally, experimentally induced nostalgia can enhance self-reported meaning in life and bolster positive emotions [71,72]. Similar to positive contemplation of the past through nostalgia, positive thinking about the future corresponded to enhanced meaning. The frequency of thoughts about future legacy is also related to meaning in life [73-75].

In this study, the correlational and mediation analyses found that future-time words were only related to the frequency of insightful words, not causal words. This suggests that meaning-making about the future involves causal reasoning less often and insightful reasoning more often. The constructive episodic simulation hypothesis posits that future mental time travel focuses more on the completeness of event narratives, thus favoring insightful reasoning [76]. Simultaneously, we also found that the direct effect of past mental time travel on positive emotions varied in both samples. This suggests that the meaning-making process plays a very important role (Park [27] and Ord et al [50]). Given the key role of mental time travel in meaning-making and promoting positive emotions, further research is needed to explore this mechanism [77].

### Theoretical and Practical Implications

This study explored the adaptive function of meaning-making in stressful situations by integrating a cyclical model of stress resilience and stress-related growth with the meaning-making model [27,50]. This validates the mediating role of meaning-making between stress and positive emotions. Under stress, individuals engage in positive reappraisal to construct meaning from stressful events. Subsequently, through mental time travel, seeking and assigning meaning, individuals promote positive emotions under stress.

Second, this research introduces the method of mini–meta-analysis in a large-sample study to investigate the establishment of mediation effects, contributing to resolving the replication crisis. Given the vast amount of data obtained from Weibo crawlers, large sample sizes can lead to false positives. Therefore, a combined meta-analysis of multiple effect sizes in the same study would be particularly useful. Regardless of the number of studies conducted, researchers can succinctly summarize the results of each study through a meta-analysis. Researchers suggested that journal peer reviewers and editors should encourage authors to perform mini–meta-analyses on their own research results to determine the stability and consistency of the study effects and that conducting meta-analyses on papers can improve the precision of estimates (ie, narrow confidence intervals) [78,79]. Conducting mini–meta-analyses of a few studies can help researchers understand and strengthen their conclusions.

Third, from a practical perspective, this study underscores the need to recognize the positive aspects of stress and prevent the demonization of stress in the “stress is debilitating” narrative. Because stress cannot be eradicated or entirely avoided, finding ways to coexist with and grow from a psychological standpoint is crucial for long-term strategies. Mental time travel, an effective way to elicit meaning-making, can be developed as an intervention method to promote better coping with stress in individuals [80-82].

### Limitations and Future Directions

This study had certain limitations. First, the cyclical model of stress resilience and stress-related growth discusses how appraisals, effective coping strategies, and personal resources modulate an individual’s stress response [50]. However, existing research has only explored meaning-making as an effective strategy. Future research should examine whether and how other coping strategies work in conjunction with meaning-making to influence positive poststress emotions.

Second, the expressions of stress, meaning-making, and positive emotions explored in this study’s Weibo analysis were essentially correlated. However, the model established was theory-based, and the data were not accessed at different time points. Future research should benefit from more laboratory designs that manipulate mental time travel to observe changes in meaning-making and positive emotions. Alternatively, momentary ecological assessment methods in daily settings and longitudinal studies can be used to obtain robust results.

Finally, the focus of this study was on whether mental time travel words can predict positive emotion words through meaning-making words; however, no distinction was made between categories of positive emotion words. Moreover, big data studies face the challenge of being unable to accurately manipulate or measure subtle differences in meaning while acquiring massive amounts of data [83]. Therefore, more refined studies should be designed in the future to validate the findings of big data studies and provide more reliable evidence.

### Conclusions

This study concludes that individuals can adapt positively to stress by engaging in meaning-making processes that are triggered by mental time travel and reflected in their social media posts. The study’s mediation model confirmed that mental time travel leads to meaning-making, which fosters positive emotional responses to stress. This finding is significant because it demonstrates the potential of mental time travel as a psychological strategy to facilitate positive adaptation to stressful situations.

## Appendix

Multimedia Appendix 1 Additional tables.

## Abbreviations

LIWC: Linguistic Inquiry and Word Count

## Data Availability

The datasets generated during or analyzed during this study are available in the (Figshare) repository [84].
