# Supplementary material for: The Mediating Role of Meaning-Making in the Relationship Between Mental Time Travel and Positive Emotions in Stress-Related Blogs: Big Data Text Analysis Research
Source: J Med Internet Res. 2025 Feb 21;27:e63407. doi: 10.2196/63407 (PMC11890143; doi:10.2196/63407)
Supplement: Multimedia Appendix 1 [file jmir_v27i1e63407_app1.docx]

Table S1. Word frequency table of high-frequency words for word frequency analysis of microblog posts (top 60)

| No. | Text | Frequency | Number | Text | Frequency | Number | Text | Frequency |
| --- | --- | --- | --- | --- | --- | --- | --- | --- |
| 1 | Self | 4491 | 21 | If | 762 | 41 | Kid | 607 |
| 2 | No | 2024 | 22 | Time | 760 | 42 | A little | 598 |
| 3 | We | 1692 | 23 | May | 759 | 43 | Big | 578 |
| 4 | Reason | 1587 | 24 | Try | 745 | 44 | Happiness | 569 |
| 5 | Livelihood | 1535 | 25 | Everyone | 737 | 45 | Question | 548 |
| 6 | Can | 1439 | 26 | So | 725 | 46 | Activity | 547 |
| 7 | Moment | 1326 | 27 | They | 712 | 47 | And | 535 |
| 8 | Work | 1230 | 28 | Thing | 708 | 48 | Can’t | 531 |
| 9 | Feel | 1190 | 29 | Other | 692 | 49 | Friend | 507 |
| 10 | Now | 1179 | 30 | Everyday | 682 | 50 | Don’t know | 496 |
| 11 | Because | 1136 | 31 | Actually | 669 | 51 | Tomorrow | 491 |
| 12 | Don’t | 1084 | 32 | Won’t | 666 | 52 | Fighting | 490 |
| 13 | But | 1066 | 33 | Many | 666 | 53 | See | 470 |
| 14 | This | 970 | 34 | Together | 657 | 54 | Need | 468 |
| 15 | Today | 951 | 35 | Good night | 652 | 55 | One day | 468 |
| 16 | Hope | 943 | 36 | Always | 649 | 56 | Although | 464 |
| 17 | Feelings | 909 | 37 | Such | 636 | 57 | Know | 463 |
| 18 | Recently | 869 | 38 | Already | 635 | 58 | Withstand | 460 |
| 19 | How | 829 | 39 | Start | 626 | 59 | Emotion | 447 |
| 20 | Happy | 764 | 40 | Like | 614 | 60 | Life | 438 |

Table S2. Descriptive statistics and correlation analysis of main variables for Sample 1 text analysis (*n* = 165,374)

|  | *M (%)* | *SD* | 1 | 2 | 3 | 4 | 5 | 6 |
| --- | --- | --- | --- | --- | --- | --- | --- | --- |
| 1 Rate of positive emotions words | 0.10 | 0.004 | 1.00 |  |  |  |  |  |
| 2 Rate of insightful words | 0.06 | 0.002 | .07^***^ | 1.00 |  |  |  |  |
| 3 Rate of causal words | 0.03 | 0.001 | .03^***^ | .10^***^ | 1.00 |  |  |  |
| 4 Rate of past time words | 0.01 | 0.002 | .01^**^ | .03^***^ | .01^***^ | 1.00 |  |  |
| 5 Rate of present time words | 0.01 | 0.001 | .02^***^ | .03^***^ | .07 | .03^***^ | 1.00 |  |
| 6 Rate of future-time words | 0.01 | 0.001 | .03^***^ | .01^***^ | -0.01 | .01^**^ | .03^***^ | 1.00 |

Note: ***p* < .01; *** *p* < .001.

Table S3. Descriptive statistics and correlation analysis of main variables for Sample 2 text analysis (*n* = 166,337)

|  | *M (%)* | *SD* | 1 | 2 | 3 | 4 | 5 | 6 |
| --- | --- | --- | --- | --- | --- | --- | --- | --- |
| 1 Rate of positive emotions words | 0.10 | 0.004 | 1.00 |  |  |  |  |  |
| 2 Rate of insightful words | 0.06 | 0.019 | .07^***^ | 1.00 |  |  |  |  |
| 3 Rate of causal words | 0.03 | 0.001 | .02^***^ | .10^***^ | 1.00 |  |  |  |
| 4 Rate of past time words | 0.01 | 0.003 | .01 | .02^***^ | .02^***^ | 1.00 |  |  |
| 5 Rate of present time words | 0.01 | 0.007 | .01^**^ | .02^***^ | .01^***^ | .04^***^ | 1.00 |  |
| 6 Rate of future-time words | 0.01 | 0.005 | .02^***^ | .03^***^ | -.01 | .01 | .04^***^ | 1.00 |

Note: ***p* < .01; *** *p* < .001.

Table S4. Mediation models

| Sample | Mental time travel | Meaning-making | Positive emotions | a path | b path | c path | Indirect effect | 99% CI |
| --- | --- | --- | --- | --- | --- | --- | --- | --- |
| 1 | Rate of future-time words | Rate of insightful words | Rate of positive emotions words | 0.04*** | 0.17*** | 0.23*** | 0.008 | [0.002, 0.021] |
| 2 | Rate of future-time words | Rate of insightful words | Rate of positive emotions words | 0.10*** | 0.17*** | 0.14*** | 0.017 | [0.007, 0.037] |
| 1 | Rate of future-time words | Rate of causal words | Rate of positive emotions words | -0.01 | 0.11*** | 0.24*** | -0.002 | [-0.005, -0.001] |
| 2 | Rate of future-time words | Rate of causal words | Rate of positive emotions words | -0.01 | 0.07*** | 0.16*** | -0.001 | [-0.002, 0.001] |
| 1 | Rate of past time words | Rate of insightful words | Rate of positive emotions words | 0.18*** | 0.17*** | 0.08* | 0.030 | [0.016, 0.052] |
| 2 | Rate of past time words | Rate of insightful words | Rate of positive emotions words | 0.11*** | 0.17*** | -0.01 | 0.019 | [0.007, 0.041] |
| 1 | Rate of past time words | Rate of causal words | Rate of positive emotions words | 0.05*** | 0.11*** | 0.10** | 0.005 | [0.001, 0.025] |
| 2 | Rate of past time words | Rate of causal words | Rate of positive emotions words | 0.09*** | 0.07*** | 0.01 | 0.006 | [-0.001, 0.025] |

Note: **p* < .05, ***p* < .01, ****p* < .001; a indicates the path from mental time travel to meaning-making; b indicates the path from meaning-making to positive emotions; c indicates the path from mental time travel to positive emotions

Table S5. Words list of Category Linguistic Processes Used in the study

| Category Linguistic Processes | Abbreviation | Examples |
| --- | --- | --- |
| Affect processes | affect |  |
| Positive emotion | posemo | love, nice, sweet |
| Cognitive processes | cogproc |  |
| Insight | insight | think, know, consider |
| Causation | casue | because, effect, hence |
| Time orientations | TimeOrient |  |
| Past focus | past | ago, did, talked |
| Present focus | present | today, is, now |
| Future focus | future | may, will, soon |
